# The Roots of Neurodegenerative Disease Found in Developmental Hematopoiesis

**DOI:** 10.1097/HS9.0000000000000010

**Published:** 2017-12-20

**Authors:** Michael D. Milsom

**Affiliations:** 1 Division of Experimental Hematology, German Cancer Research Center (DKFZ); 2 Heidelberg Institute for Stem Cell Technology and Experimental Medicine (HI-STEM), Heidelberg, Germany

**Figure 1:**
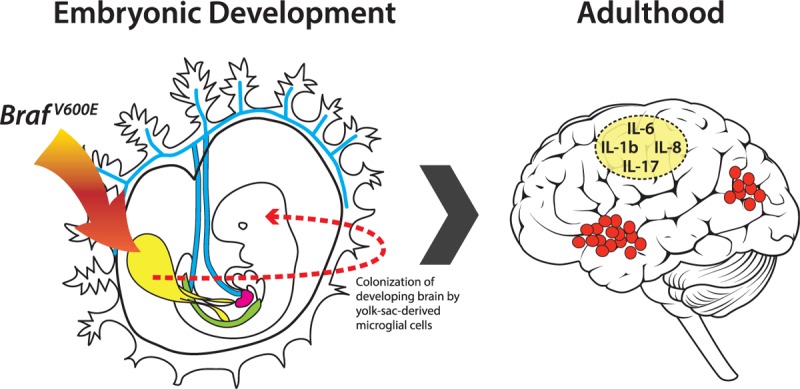
Braf^V600E^ mutations acquired within erythro-myeloid progenitors in the yolk-sac during early development are then inherited by their progeny, the tissue-resident macrophages, such as microglial cells in the brain. Mutant Braf microglial cells are capable of clonal expansion in the adult brain and can then drive the evolution of neurodegenerative disease, likely via the production of neurotoxic inflammatory cytokines, T-cell infiltration, phagocytosis, and extracellular matrix remodeling. Figure adapted in part from K. A. Mikkola, Stuart H. Orkin, Development 2006;133:3733–3744; doi: 10.1242/dev.02568.

Late-onset neurodegenerative diseases evolve over the course of many years and have complex etiologies, with the exact pathophysiology of a given disease likely being driven by multiple inherited and environmental factors. In a recent letter published in *Nature*, Drs Prinz, Abdel-Wahab, and Geissmann led a program of research which aimed to explore the role of BRAF-mutated microglial cells in mediating the onset of this disease.^1^ The rationale for studying this particular mutation within microglial cells, the tissue-resident macrophage population found in the brain, was that several previous studies had noted that patients with histiocytoses had evidence of neurodegeneration.^2,3^ Histiocytoses result from a clonal expansion of tissue-resident macrophages that have acquired somatic mutations aberrantly activating the RAS/RAF/MEK/ERK signaling pathway. These include the BRAF(V600E) mutation, which is a well characterized oncogenic mutation across several different forms of solid tumor, and can drive the emergence of leukemia—but never neurodegeneration—if it is acquired within the definitive hematopoietic stem cell compartment. However, microglial cells are not predominantly derived from adult hematopoietic stem cells, but are rather generated during embryonic development from yolk-sac-derived erythro-myeloid progenitors that occur during the first waves of primitive hematopoiesis.^4,5^ Therefore, a possible direct link between the BRAF(V600E) mutation and neurodegenerative disease might exist if this mutation was acquired within somatic cells of the developing yolk-sac, which then went on to colonize the developing brain as microglial cells (Fig. 1).

In order to test this hypothesis in the mouse, Mass and colleagues used a tamoxifen-inducible Cre recombinase, driven by the *Csf1* receptor locus, to delete LoxP-flanked stop cassettes upstream of *Braf*^*V600E*^ and *Rosa26*^*YFP*^ alleles in yolk-sac erytho-myeloid progenitors.^1^ Injection of pregnant female mice with 4-hydroxytamoxifen at day E8.5 of development led to the subsequent generation of viable offspring that demonstrated mosaic expression of both Braf^V600E^ and YFP in tissue-resident macrophages, including microglia, but not in bone marrow-derived progeny. These mutant resident macrophages demonstrated a modest increase in proliferation rate and, accordingly, were found at a higher frequency within mouse tissues compared with their counterparts in control mice harboring the Cre-inducible YFP reporter but no mutant *Braf* allele. Despite this clonal expansion-like phenotype, mice did not develop any form of malignancy. However, longitudinal analysis of the mice revealed the evolution of a progressive neurodegenerative disease. Starting from the age of 4 to 6 months, adult mice presented with significantly reduced gross motor skills and a unilateral loss of hindlimb reflexes, with approximately 90% of experimental mice demonstrating symptoms of neurological disease by 7 months of age. By 9 months of age, the disease had progressed to the extent that around 60% of the *Braf*-mutant mice had full paralysis. This phenotype was consistent with pathological changes in the brain that were analogous to those seen in patients with neurodegenerative disease. Mutant microglial cells with activated ameboid morphology were found in large clusters in the thalamus, brain stem, cerebellum, and spinal cord. In the areas of the brain where these clusters were located, there was also evidence of extensive synaptic and neuronal loss, demyelination, and the deposition of amyloid precursor protein. Gene expression analysis of YFP^+^ microglial cells isolated from paralyzed mice confirmed the aberrant activation of RAS-associated signaling pathways, and also revealed increased expression of genes corresponding to inflammatory response signatures, transcripts associated with phagocytosis, and matrix-associated genes. These gene expression changes are likely representative of biological processes that mediate the neurodegenerative pathology observed in the mice.

In an effort to link hyper-activation of Braf signaling within microglial cells to the neurodegenerative disease observed in mice, the authors then went on to chronically treat mice with the BRAF inhibitor, PLX4720, starting at either 1 or 3 months of age. Treatment decreased MEK/ERK signaling in mutant microglial cells in vivo and resulted in decreased proliferation and a modest reduction in the abundance of microglial cells in older mice. Perhaps most importantly, early intervention with the BRAF inhibitor decreased the severity of symptoms associated with loss of motor skill coordination and delayed the age of onset of the phenotype, while both treatment regimens significantly inhibited the progression of the disease. Not only does this demonstrate the requirement for Braf signaling in the development and progression of the disease, but it also suggests that this may be a novel therapeutic target to treat certain forms of neurodegeneration.

Finally, the authors were able to validate their findings in the above mouse model by analyzing brain tissue from a small number of patients with histiocytoses carrying a BRAF mutation. Analysis of the histology of brain sections from 3 patients with Erdheim–Chester disease demonstrated the accumulation of activated microglial cells with nuclear pERK staining located at sites of neuronal loss, astrogliosis and demyelination. Gene expression analysis of brain tissue from a patient with Langerhans cell histiocytosis and a patient with juvenile xanthogranuloma revealed similar changes in inflammatory, phagocytic, and matrix remodeling transcriptional signatures to those observed in the mouse model.

Taken together, these data strongly support the role of microglial cells in initiating and propagating neurodegeneration, and also demonstrate that the acquisition of the somatic Braf^V600E^ mutation during early development in the yolk-sac has a very different disease outcome to the oncogenic effects observed when this mutation is acquired in adult tissues. The demonstration of the effectiveness of BRAF inhibition on disease progression also highlights the potential translational application of these findings, although challenges that would need to be met in the future include determining how broadly applicable this mechanism is and developing a screening approach that would allow the earliest possible intervention, possibly before the clinical symptoms of histiocytosis become apparent.
